# Medicinal Plants in COVID-19: Potential and Limitations

**DOI:** 10.3389/fphar.2021.611408

**Published:** 2021-03-24

**Authors:** Xin Yi Lim, Bee Ping Teh, Terence Yew Chin Tan

**Affiliations:** Herbal Medicine Research Centre, Institute for Medical Research, National Institutes of Health, Ministry of Health Malaysia, Shah Alam, Malaysia

**Keywords:** complementary therapy, ethnopharmacology, COVID-19, coronavirus, medicinal plants, herbs

## Abstract

Currently, the search to identify treatments and vaccines for novel coronavirus disease (COVID-19) are ongoing. Desperation within the community, especially among the middle-and low-income groups acutely affected by the economic impact of forced lockdowns, has driven increased interest in exploring alternative choices of medicinal plant-based therapeutics. This is evident with the rise in unsubstantiated efficacy claims of these interventions circulating on social media. Based on enquiries received, our team of researchers was given the chance to produce evidence summaries evaluating the potential of complementary interventions in COVID-19 management. Here, we present and discuss the findings of four selected medicinal plants (*Nigella sativa*, *Vernonia amygdalina*, *Azadirachta indica*, *Eurycoma longifolia*), with reported antiviral, anti-inflammatory, and immunomodulatory effects that might be interesting for further investigation. Our findings showed that only *A. indica* reported positive antiviral evidence specific to the severe acute respiratory syndrome coronavirus 2 (SARS-CoV-2) based on preliminary *in silico* data while all four medicinal plants demonstrated differential anti-inflammatory or immunomodulatory effects. The definitive roles of these medicinal plants in cytokine storms and post-infection complications remains to be further investigated. Quality control and standardisation of medicinal plant-based products also needs to be emphasized. However, given the unprecedented challenges faced, ethnopharmacological research should be given a fair amount of consideration for contribution in this pandemic.

## Introduction

The emergence of a new coronavirus, known as the SARS-CoV-2 has initiated a pandemic of COVID-19 (World Health Organisation, 2020b). More than 31 million infections with at least 960,000 COVID-19 associated deaths were reported by September 23, 2020 (World Health Organisation, 2020c). Since its first reported case in Wuhan, China in December 2019 (World Health Organisation, 2020c), new discovered evidence by both clinicians and researchers globally have helped shed some light on the disease pathogenesis and the nature of the virus itself. The availability of new information subsequently fed policy changes on transmission prevention strategies as well as development of preventative vaccines and therapeutic drug candidates. Enforced physical distancing, hand hygiene, and arguably proper usage of personal protective equipment including wearing a surgical mask remains the most effective way of controlling the spread of the disease, with most countries which adopted such measures reporting some success in curbing the disease spread (Chu et al., 2020; Sardar et al., 2020). However, several challenges remain in maintaining these drastic measures of enforced physical distancing for long periods of times. Resurgences of infection waves were reported in few countries after the relaxation of rules. In addition, the economic impact of prolonged lock down on social issues such as loss of income and increased poverty, especially for the low and middle-income countries, is evident (Bonaccorsi et al., 2020; United Nations Development Programme, 2020).

**FIGURE 1:**
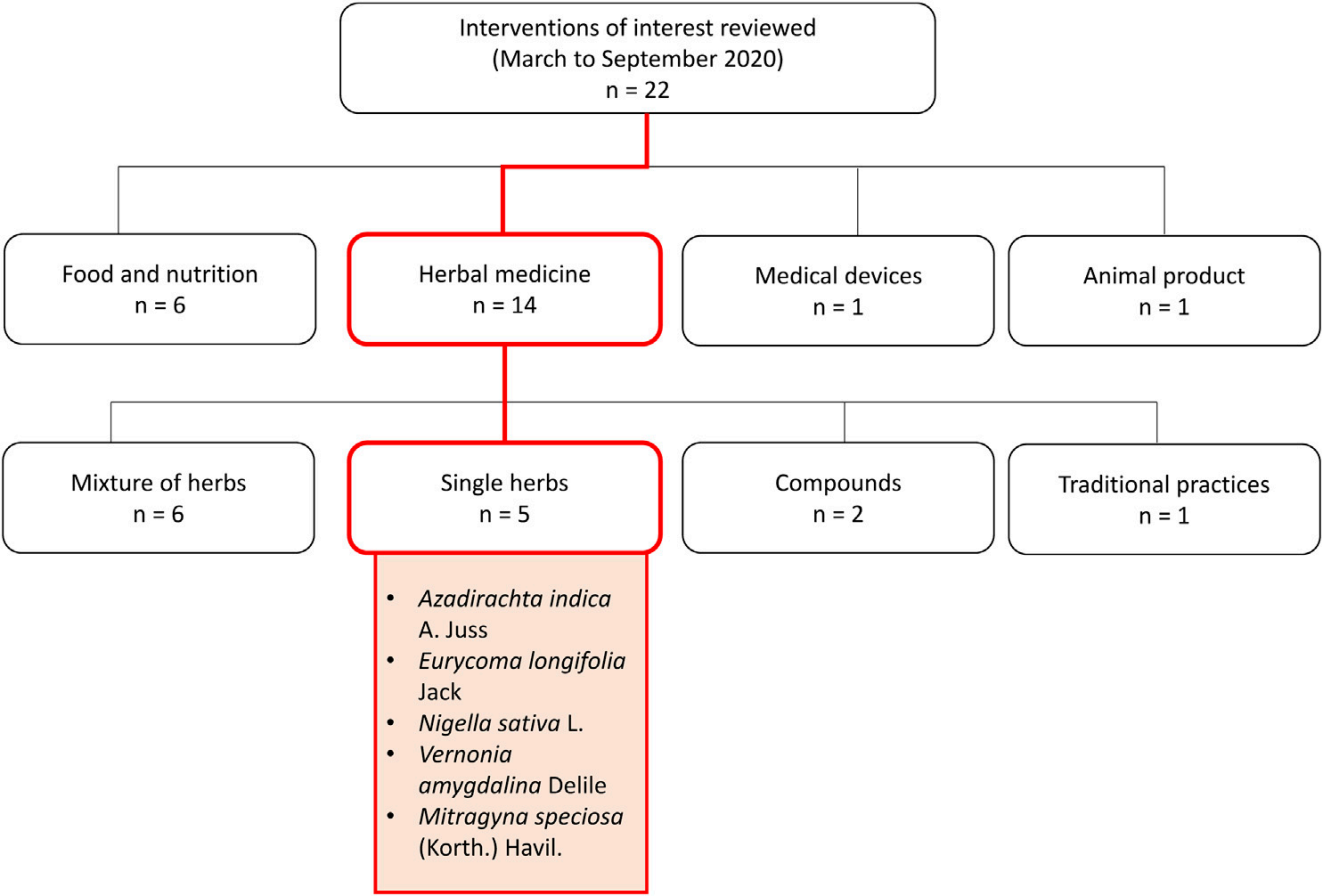
Selection of single medicinal plants as interventions of interest.

As the world looks towards science in search of an effective drug or vaccine, a few countries, such as China and India, with long histories of traditional medicine use (Li et al., 2020; Rastogi et al., 2020), have also started exploring the role of traditional and complementary, alongside conventional treatment. The Malaysian community, coming from a tropical multi-racial country rich in flora and fauna, also appears to be interested in venturing towards the use of herbal and complementary medicine, some of which are based on local traditional knowledge. During the Movement Control Order implemented by the Malaysian Government in March 2020 in attempts to curb the disease spread, the herbal medicine research arm of biomedical research institute in Malaysia has received numerous queries on the potential use of complementary remedies including single medicinal plants, traditional remedies, finished herbal products, supplements, food products, and medical devices against COVID-19. These queries were mainly submitted directly by the general public and persons with readily available herbal products, or identified through highly circulated messages on several social media platforms. From March to September 2020, 22 interventions of interest were reviewed through searches conducted on electronic databases such as PubMed, Web of Science, Google Scholar; as well as hand searching of grey literature, including books on herbal and traditional medicine available in institutional library resources. The predetermined search terms used are ‘COVID-19’, ‘antiviral’, ‘anti-inflammatory’, ‘immune system’, ‘immunomodulatory’, ‘safety’, ‘toxicity’, in combination with the name of the main intervention of interest or its synonyms. From these evidence summaries, five were single medicinal plants including *Azadirachta indica* A. Juss, *Eurycoma longifolia* Jack, *Nigella sativa* L., *Gymnanthemum amygdalinum* (Delile) Sch. Bip. (or *Vernonia amygdalina* Delile), and *Mitragyna speciosa* (Korth.) Havil (Figure 1).

Of the five individual medicinal plants, this review presents and discussed the available evidence of four selected plants (A. *indica*, *E. longifolia*, *N. sativa*, and *V. amygdalina*), considering their efficacy evidence as antiviral, anti-inflammatory, and immunomodulatory agents for use in COVID-19 management; as well as completeness of quality and safety data to be incorporated into human trials. *M. speciosa* was not further discussed here due to established reports on toxicity and dependance (Meireles et al., 2019). *M. speciosa* is also currently listed as a prohibited ingredient in natural products in Malaysia (National Pharmaceutical Regulatory Agency, Ministry of Health Malaysia, 2020). Although public interest in the use of the selected four medicinal plants (*A. indica*, *E. longifolia*, *N. sativa*, and *V. amygdalina*) for COVID-19 seemed strong, there are concerns on their efficacy and safety. As research in COVID-19 treatment intensifies, exploring the potential roles of medicinal plants, lobbying and extrapolating from known scientific evidence on safety and efficacy, can be beneficial.

## Medicinal Plants in COVID 19: Efficacy, Safety, and Research Gaps

In the research of phytomedicine, it is common to observe multiple pharmacological properties from a single plant. It is now well understood that a single plant may contain a wide range of phytochemicals, making ethnopharmacology research both full of possibilities yet challenging (Süntar, 2019). Overall, these selected interventions of interest discussed here can be broadly categorised into those with 1) antiviral, 2) anti-inflammatory, 3) immunomodulatory effects, and more often 4) a combination of these effects, based on available evidence for efficacy (Table 1). Details on quality, efficacy, and safety of individual studies is presented in Supplementary Table S1. On top of exhibiting direct antiviral effects, medicinal plants with reported anti-inflammatory activities may have pleiotropic roles in COVID-19 management as the elevation of inflammatory markers such as interleukin (IL)-6, erythrocyte sedimentation rate (ESR), and C-reactive protein (CRP) has been associated with severe disease with worse outcomes among COVID-19 patients, most likely related to cytokine storm (Zeng et al., 2020).

**TABLE 1:** Pharmacological properties and safety evidence of selected herbs and supplements (Koley and Lal, 1994; Talwar et al., 1995; Talwar et al., 1997; Badary et al., 1998; Hore et al., 1999; Salem and Hossain, 2000; Petrovsky, 2006; Bamosa et al., 2010; Datau et al., 2010; Iyyadurai et al., 2010; Momoh et al., 2010; Salem et al., 2011; Schumacher et al., 2011; Venugopalan et al., 2011; Aljindil, 2012; Choudhary et al., 2012; Momoh et al., 2012; Abdel-Moneim et al., 2013; Li et al., 2013; Mishra and Dave, 2013; Saalu et al., 2013; Adedapo et al., 2014; Nabukenya et al., 2014; Tran et al., 2014; Ulasli et al., 2014; Yee et al., 2014; Gholamnezhad et al., 2015; Majdalawieh and Fayyad, 2015; Ashfaq et al., 2016; George et al., 2016; Im et al., 2016; Omoregie and Pal, 2016; Rehman et al., 2016; Zakaria et al., 2016; Onasanwo et al., 2017; Salem et al., 2017; Tavakkoli et al., 2017; Asante et al., 2019; Onah et al., 2019; Ruan et al., 2019; Borkotoky and Banerjee, 2020; Dwivedi et al., 2020).

		*Azadirachta indica* A. Juss.	*Eurycoma longifolia* Jack	*Nigella sativa* L.	*Gymnanthemum amygdalinum* (Delile) Sch. Bip.
Pharmaco-logical properties	Antiviral	+^a^		++	
	Anti-inflammatory	+	+	++	+
	Immuno-modulatory	++	++	++	++
Safety	Preclinical	Leaf extract: arrhythmia, hypoglycemia, and blood pressure reduction	Standardised aqueous extract (root): no-observed-adverse-effect-level (NOAEL) dose >1,000 mg/kg orally; minimal mammalian carcinogenicity; no genotoxicity	Thymoquinone: hypoglycaemia and hepatic impairment	Aqueous extract (Leaf): kidney congestion;
		Seed oils and extracts: abortifacient			Ethanolic extract (Leaf): testicular toxicity
	Clinical	Seed oils and extracts: acidosis, renal injury, anti-human chorionic gonadotropin effects	Safe dose (standardised aqueous extract) used in clinical trial: 200 mg/day	Seed: Safe up to 3 months of consumption	

+: positive preclinical evidence published; ++: positive clinical evidence published.

^a^ COVID-19 specific evidence.

According to our evidence summaries, *N. sativa* (black cumin) seed was among one of the medicinal plants with most published positive evidence. Ethanolic extracts of *N. sativa* seeds demonstrated antiviral properties by decreasing viral load, alpha fetoprotein, and improved liver function parameters among hepatitis C infected patients (Abdel-Moneim et al., 2013). In animal studies, *N. sativa* seed oil presented both antiviral and immunomodulatory effects against cytomegalovirus, reducing viral loads to an undetectable level. It can also enhance the immune response by increasing CD3 and CD4 counts, as well as up-regulating interferon-gamma (IFN-γ) release from Natural Killer T-cells and macrophages (Salem and Hossain, 2000). In cell studies, ethanolic extracts of *N. sativa* seeds also demonstrated inhibitory activity against coronavirus species MHVA59 (mouse hepatitis virus-A59) replication by downregulating gene expressions of various leukocyte transient receptor proteins (TRP) such as the TRPA1, TRPC4, TRPM6, TRPM7, TRPM8 and TRPV4 genes (Ulasli et al., 2014). Traditionally, *N. sativa* is used for a diverse range of indications including in respiratory diseases such as asthma (Al-Jauziyah, 2003). The benefits of *N. sativa* supplementation (details of formulation unclear) in improving asthmatic symptoms have also been reported in a clinical trial, and is thought to be partially due to the anti-hypersensitivity and potentially anti-inflammatory properties (Salem et al., 2017). Positive preclinical and clinical evidence of *N. sativa*’*s* immunomodulatory and anti-inflammatory effects have been collectively concluded in three separate review papers (Gholamnezhad et al., 2015; Majdalawieh and Fayyadet, 2015; Tavakkoli et al., 2017). More interestingly about *N. sativa* and its bioactive compound thymoquinone, is their immunomodulatory effects reported in respiratory diseases, including those of infectious origin. An *in vitro* study has reported that thymoquinone enhances survival of antigen-activated CD8^+^ cells, highlighting the potential for adoptive T-cell therapies (Salem et al., 2011). The potential of *N. sativa* to modulate B cell-mediated immune response while balancing Th1/Th2 ratio to potentiate T cells-mediated immune response merits further investigation. This activity can be explored as an adjunct to potential vaccine candidates to mediate meaningful and sustained immune response post vaccination (Petrovsky, 2006), which is one of the main challenges with current potential COVID-19 vaccines in development (Sahin et al., 2020). As for safety, long-term consumption (up to three months) of *N. sativa* seeds at 3 g/day in humans reported no significant adverse effects on both liver and kidney functions (Bamosa et al., 2010; Datau et al., 2010). However, precautions should be paid towards thymoquinone as animal toxicity studies at high doses of 2–3 g/kg have resulted in hypoglycaemia and hepatic enzyme derangements (Badary et al., 1998).

Another plant that has shown immune enhancing effects as adjunct to vaccines is *G. amygdalinum*, more commonly known as *V. amygdalina* or bitter leaf. This plant was reported to be traditionally used to relieve fever, diarrhoea, cough, and headache (Plant Resources of Tropical Africa, PROTA, 2004). Aqueous extracts of G. *amygdalinum* showed positive effects in enhancing immune response by increasing the levels of white blood cells and CD4^+^ (Momoh et al., 2010; Momoh et al., 2012; Im et al., 2016). With the capability to increase the CD4^+^ cell counts, this extract was reported to be adjuvant to antiretroviral therapy in HIV positive patients (Momoh et al., 2012). In addition, the aqueous extract also demonstrated potential immune augmenting effects as adjuvant to Hepatitis B vaccine by increasing levels of surface antigen of the Hepatitis B virus (rHBsAg)-specific antibodies immunoglobulin M, immunoglobulin G sub class 1, and immunoglobulin A (Onah et al., 2019). As a plant with various phytochemicals with the potential to exhibit multimodal mechanism of actions, ethanol, methanol, and acetone extracts also reported anti-inflammatory activity in laboratory animals via modulation of levels of inflammatory cytokines and mediators including the pro-inflammatory (prostaglandin-endoperoxide synthase 2, nuclear factor kappa B (NFκB), tumor necrosis factor-alpha (TNF-α), IL-1β, IL-6, IL-8, nitric oxide, CRP) and anti-inflammatory markers (Adedapo et al., 2014; Omoregie and Pal, 2016; Onasanwo et al., 2017; Asante et al., 2019). Despite the reported potent activity of this plant in regulating the immune and inflammation responses, its toxicity profile remains be ascertained. Although no mortality was reported in an acute toxicity study in animals (Zakaria et al., 2016), subacute administration of the aqueous extract (200 and 600 mg/kg body weight) in rats caused kidney congestion (Nabukenya et al., 2014) while testicular toxicities was reported with an ethanol extract (300 and 600 mg/kg) (Saalu et al., 2013). Currently, there is insufficient direct evidence on the efficacy of *V. amygdalina* in COVID-19, despite various reported antiviral, anti-inflammatory, and immunomodulatory effects.

The leaves of neem (*A. indica*), a popular Indian plant, is traditionally boiled and consumed for treatment of fever (Burkill, 1935), with reported anti-inflammatory effects in animal studies (Schumacher et al., 2011). *In vitro* and *in silico* docking studies demonstrated that neem leaves extracts and its phytochemicals such as flavonoids and polysaccharides have direct antiviral effects against various viruses including dengue (Dwivedi et al., 2020) and Hepatitis C Virus (Ashfaq et al., 2016). Specific to SARS-CoV-2, molecular docking studies have demonstrated that the neem derived compounds nimbolin A, nimocin, and cycloartanols have the potential to bind to envelope (E) and membrane (M) glycoproteins of the SARS-CoV-2 and act as inhibitors (Borkotoky and Banerjee, 2020). As for immunomodulatory effects, both neem seeds and leaves reported positive effects in enhancing immune response in animals (Venugopalan et al., 2011; Aljindil, 2012). In mice vaccinated with *Brucella* Rev-1 vaccine, neem seed extract given subcutaneously enhanced the production of IFN-γ post vaccination (Aljindil, 2012). However, the main issue with exploring neem’s potential for COVID-19 is its safety profile. Although neem leaves have been used traditionally for a long time, well documented safety records are still insufficient. Several animal toxicity studies have reported variable adverse effects including arrhythmia, hypoglycaemia, and blood pressure reduction at high doses of neem leaf extracts (Koley and Lal, 1994; Hore et al., 1999). Human cases of acidosis and renal injury have also been reported on neem seed oil consumption (Iyyadurai et al., 2010; Mishra and Dave, 2013). In pregnant women, neem seed extracts should be avoided as animal studies have shown its abortifacient effects (Talwar et al., 1997) while human trials have reported its anti-human chorionic gonadotropin effects (Talwar et al., 1995). That being said, the traditional use of neem for medicinal purposes is largely focused on leaves consumption, boiled in water and drank (Mustapha et al., 2017). In view of safety concerns, studies establishing safe doses of neem leaves specific to the formulation intended for use is required prior to further investigations on efficacy.

The main challenges of phytopharmaceutical development for therapeutic claims is quality control, identification, and standardisation of the bioactive compounds of a plant-based product. Due to the inherent nature of natural products containing multiple bioactive and chemical markers, the quality control process to meet stringent regulatory standards of safety considerations is time consuming and lengthy (Tan et al., 2020). Tongkat Ali, or *E. longifolia*, a popular Malaysian plant traditionally used for improving men’s health (Rehman et al., 2016) is among one of the few natural products with established standardisation and safety data available. Acute, subacute, and subchronic toxicity studies of the powdered root of *E. longifolia* in rats reported a calculated acceptable daily intake of up to 1.2 g/adult/day in humans (Li et al., 2013). Safety assessment of the standardised aqueous extract of *E. longifolia* (acute, subacute, and 90 days subchronic general toxicity studies) conducted according to the relevant Organisation for Economic Co-operation and Development (OECD) guidelines reported no toxic effects in rats (Choudhary et al., 2012). Specific toxicity studies of the same extract also reported low mammalian mutagenicity with no genotoxic effects (Yee et al., 2014). Although no direct antiviral effects were reported with standardised aqueous extract of *E. longifolia*, clinical data have shown its positive effects in enhancing immune response in the aging population by improving the CD4^+^ counts, with a safe dose of 200 mg/day (George et al., 2016). Preclinical evidence of the anti-inflammatory properties of *E. longifolia* are also available. Among the potential bioactive anti-inflammatory compounds isolated from *E. longifolia* include eurycomalactone, 14,15β-dihydroklaieanone, and 13,21-dehydroeurycomanone with potent NF-κB inhibitory effects (Tran et al., 2014). Several phenolic compounds isolated from the roots of *E. longifolia* were also reported to significantly reduce expression of IL-6 in lipopolysaccharide stimulated RAW264.7 macrophage (Ruan et al., 2019). Given its well established safety profile, future investigations on the potential anti-inflammatory effects of *E. longifolia* may be explored in the context of COVID-19. However, as many of the published studies were industrial sponsored (Choudhary et al., 2012; Yee et al., 2014; George et al., 2016), the potential for bias remains to be ascertained.

## Perspective: Developing Herbal Medicine for COVID-19

One year into the pandemic, it has become apparent that developing an effective antiviral against the SARS-CoV-2 is challenging due to the virus infectivity and disease course. Viral life cycle modelling studies suggested that early administration of a highly potent antiviral is needed to effectively curb the infection and preserve host cells. This number also coincides with the average number of days for peak viral load to occur and symptoms onset, making it a challenge for timely administration of antivirals in community spreading (Goncalves et al., 2020). Though there have been many claims on antimicrobial properties of the selected medicinal plants discussed here, only one medicinal plant, neem, demonstrated preliminary *in silico* evidence of antiviral effects specific towards the SAR-CoV-2 (Borkotoky and Banerjee, 2020). Currently, the antiviral remdesivir is approved by the U.S Food and Drug Administration (FDA) for use in hospitalised patients with COVID-19 based on positive data from clinical trials (U.S. Food and Drug Administration, 2020). Although remdesivir improved clinical symptoms, there is insufficient evidence to support its benefits on mortality (Dyer, 2020). Remdesivir is thought to act via early termination of viral RNA synthesis hence inhibiting replication (Eastman et al., 2020). Based on viral kinetics modelling, a combination of various antivirals targeting multiple stages of the viral life cycle of infecting the host is suggested as a plausible strategy to effectively curb infection (Dodds et al., 2020). Hence, future investigations on the effects of compounds identified from neem such as nimbolin A, nimocin, and cycloartanols through a different targeted pathway (inhibition of E and M glycoproteins) (Borkotoky and Banerjee, 2020) from remdesivir may provide additional benefits.

Instead of antiviral properties, most of the medicinal plants discussed here demonstrated anti-inflammatory effects supported by *in vivo* preclinical evidence. At present, anti-inflammatory and immunomodulatory agents such as corticosteroids and IL-6 receptor antagonist are being utilised in the management of COVID-19 related cytokine storm associated with severe acute respiratory distress syndrome, in hopes to improve survival (Prescott and Rice, 2020; Saha et al., 2020). Medicinal plants such as V. *amygdalina* and *E. longifolia* demonstrated suppression effects on specific pro-inflammatory cytokines correlated with worsened COVID-19 outcome such as the IL-6 (Adedapo et al., 2014; Omoregie and Pal, 2016; Onasanwo et al., 2017; Asante et al., 2019; Ruan at al., 2019; Zeng et al., 2020). However, considering the treatment of cytokine storms are administered to patients who are severely ill, a majority of them on mechanical ventilation (Prescott and Rice, 2020), the administration of medicinal plant or an herbal formulation via the oral route will be challenging in intubated patients. Compatibility and potential of herbal formulations adsorption on nasogastric tubes also needs to be evaluated. Furthermore, as some medicinal plants such as the *E. longifolia* also reported immune-stimulating activity in older adults (George et al., 2016), the risk of worsening an existing cytokine storm needs to be evaluated. Consideration on optimal timing of administration during different disease stages to modulate the immune system is crucial to maximise the benefits versus risks of such agents (Nidadavolu and Walston, 2020; Nugraha et al., 2020). From a different perspective, it will be interesting to explore the potential role of medicinal plants with anti-inflammatory properties in post SARS-CoV-2 infection complications related to chronic inflammation such as lung fibrosis and neuropsychiatric symptoms (Fraser, 2020; Paterson et al., 2020), given the existing adverse effects associated with long term steroids use (Liu et al., 2013). As post COVID-19 complications remains a new field of study at present, investigation on long-term safety profile and pharmacokinetics of potential medicinal plants can be beneficial.

The time and processes required to develop an herbal medicine of high enough quality and consistency for therapeutic use with sufficient safety data is extremely protracted. This is due to the nature of medicinal plants containing multiple phytochemicals, which are also easily affected by agronomic factors (Süntar, 2019). In addition, identifying, isolating, and producing reference standards required for the standardisation of medicinal plants is challenging, compared to synthetic chemical entities, which are more straight forward. Standardisation of herbal products based on bioactive markers remains important to ensure batch-to-batch consistency and efficacy (Sachan et al., 2016). Due the variation in the formulations available for individual medicinal plants, adequate toxicity studies specific to the formulation of interest are required to ensure its safety (World Health Organization Regional Office for the Western Pacific, 1993). As a result of these challenges, it is highly unlikely to develop new products from scratch in time for emergency use during crises like the current COVID-19 pandemic. In times of emergency, accelerated approvals for therapeutic candidates of proven safety with minimal risk, as well as having the potential for benefits are often considered (Van Norman, 2020). These considerations drive the bulk of research to favour repurposing existing drugs, including remdesivir (Singh et al., 2020). The same concept may be applied to natural products, keeping in mind that each individual formulation and product though containing the same plant, is unique on its own. Although ideally the development of a most potentially efficacious agent is desired, in the case of considering herbal medicine for emergency use, the availability of a well-developed standardised herbal product with sufficient safety data is equally valuable. Compared to published reviews on herbal medicine in COVID-19 (Huang et al., 2020; Nugraha et al., 2020), some of the medicinal plants mentioned here including *E. longifolia* and *V. amygdalina* were not identified in previous reviews. Among the four medicinal plants reviewed here, it appears that only one (*E. longifolia*) had extensive safety data on a marketed aqueous extract to be considered for a clinical trial. However, in these individual papers, the quality data on chemical fingerprinting and quantitative assessment were not reported (Choudary et al., 2012; Yee et al., 2014; George et al., 2016). Apart from quantitative assessment of phytochemical markers and intrinsic toxicity, additional quality assessments on the risk of extrinsic toxicities from external contaminants and adulteration are also important (Posadski et al., 2013).

In these unprecedented times where the pandemic has affected people worldwide in ways unimaginable, advancing science in herbal medicine for therapeutic claims should be included as an important contribution towards research in COVID-19. As part of the efforts in strengthening science and contribution of traditional herbal medicine in the current pandemic, the World Health Organisation, with the Africa Centre for Disease Control and Prevention, and the African Union Commission for Social Affairs have recently endorsed a protocol for conducting clinical trials on herbal medicine in COVID-19 (World Health Organisation, 2020a). For the medicinal plants discussed here, in addition to requiring more direct evidence of their role in COVID-19 management, other concerns that remains to be addressed include identification of bioactive ingredients, safe dose specific to formulations, and potential drug-herb interaction prior to entering a clinical trial. Innovative ways to utilise the antimicrobial properties of medicinal plants beyond systemic absorption, such as development of medicinal plant-coated antimicrobial mask (Wang et al., 2017), can be further examined.

## Herbal Medicine Research Centre (HMRC) COVID-19 Rapid Review Team

Murizal Zainol, Siti Hajar Muhd Rosli, Adlin Afzan, Hussin Muhammad, Suganthi Jeyabalan, Mohd Ridzuan Mohd Abd Razak, Hemahwathy Chantira Kumar, Nor Azlina Zolkifli, Nor Azrina Norahmad, Muhammad Nor Farhan Sa'at, June Chelyn Lee, Elda Nurafnie Ibnu Rasid, Puspawathy Krishnan, Norfarahana Japri, Raja Nazatul Izni Raja Shahriman Shah, Nur Salsabeela Mohd Rahim, Nurmaziah Mohammad Shafie, Noorashikin Haleem, Ida Farah Ahmad, Ami Fazlin Syed Mohamed

## Conclusion

In conclusion, the four medicinal plants (*A. indica*, *E. longifolia*, *N. sativa*, and *V. amygdalina*) discussed here collectively exhibited pleiotropic effects which can potentially provide a multimodal approach via antiviral, anti-inflammatory, and immunomodulatory effects in COVID-19 management. At present, it is evidently challenging to pool data from published studies due to variation in extracts selection and a lack of well-reported standardisation data of the investigated formulations. Still, it is quite clear that there is insufficient evidence of direct antiviral effects specific to the SARS-CoV-2. Further investigations on differential anti-inflammatory and immunomodulatory effects as well as quality and safety of herbal medicines are required to ascertain their role in COVID-19 management.
