# Supplementary material for: Medicinal Plants in COVID-19: Potential and Limitations
Source: Front Pharmacol. 2021 Mar 24;12:611408. doi: 10.3389/fphar.2021.611408 (PMC8025226; doi:10.3389/fphar.2021.611408)
Supplement: Supplementary file 1 [file datasheet1.pdf]

Table S1a. Efficacy

No.	Formulation	PP	Dose	Methodology				Antiviral		Anti-inflammatory (AI) / Immunomodulatory (IM)/ Immunostimulant (IS)		Quality		Ref.
				Study model	Sample size (per group	Comparator	Ethics approval	Outcome	Study type	Outcome	Study type	PI	PA	
**A. *Gymnanthemum amygdalinum* (Delile) Sch. Bip.**														
1	50% aqueous ethanol	Leaf	300 mg/kg	Post-menopausal osteoarthritis model of female Sprague Dawley (OVX-OA) rat	8	Untreated group	Yes			**AI:** ↓ PG E2, NFκβ, IL-1β, ADAMTS-5, collagen type 10α1, caspase3. ↑ anti-inflammatory cytokine IL-10 mRNA expressions.	PC (in vivo)	No	Yes	(Madzuki et al., 2019)
2	Ethanol extract	Leaf	50 to 200 mg/kg	Sprague Dawley rat	5	Normal saline	Yes			**AI:** ↓paw oedema, antipyretic, and antinociceptive comparable to diclofenac 10mg/kg (DD).	PC (in vivo)	Yes	Yes	(Asante et al., 2019)
3	Methanol extract	Leaf	50 to 200 mg/kg	Wistar rat	6	1% Tween 80	NM			**AI:** ↓ oedema, infiltrating leukocytes protein concentration,	PC (in vivo)	Yes	No	(Onasanwo et al., 2017)

No.	Formulation	PP	Dose	Methodology				Antiviral		Anti-inflammatory (AI) / Immunomodulatory (IM)/ Immunostimulant (IS)		Quality		Ref.
				Study model	Sample size (per group	Comparator	Ethics approval	Outcome	Study type	Outcome	Study type	PI	PA	
3										malondialdehyde levels (DD).				
4	Ethanol extract	Leaf	100 mg/kg to 1,000 mg/kg	Swiss mouse	6	Untreated group	Yes			**AI:** ↓ pro-inflammatory cytokines, IFN-γ, TNF-α. (DD)	PC (in vivo)	Yes	Yes	(Omoregie et al., 2016)
5	Isolated vernoniosides A, B, C, D (four new Delta-7, 9(11)-stigmastane-type steroid saponins)	Leaf	50 µM	Murine macrophage cells RAW264.7 cells	NA	N-monomethyl-l-arginine	NA			**AI:** ↓ nitric oxide production.	PC (in vitro)	Yes	Yes	(Quasie et al., 2016)
6	Acetone extract	Leaf	100 mg/kg to 200 mg/kg	Wistar rat	5	Indomethacin	UC			**AI:** ↓ oedema, comparable to indomethacin 10mg/kg.	PC (in vivo)	Yes	UC	(Adedapo et al., 2014)
7	Ethanol extract	Leaf	200 mg/kg	Wistar rat	6	Carboxymethyl cellullose Na 2% (w/v)	NM			**AI:** ↓ pro-inflammatory IL-6. ↑ anti-inflammatory IL-10.	PC (in vivo)	Yes	No	(Setiawan et al., 2019)

No.	Formulation	PP	Dose	Methodology				Antiviral		Anti-inflammatory (AI) / Immunomodulatory (IM)/ Immunostimulant (IS)		Quality		Ref.
				Study model	Sample size (per group	Comparator	Ethics approval	Outcome	Study type	Outcome	Study type	PI	PA	
8	Aqueous extract	Leaf	50 mg/kg to 800 mg/kg	Rat	6	Distilled water	UC			**IS:** ↑ CD4+ and white blood cells count.	PC (in vivo)	No	No	(Im et al., 2016)
9	Aqueous extract	Leaf	250 mg/kg to 1,000 mg/kg	Swiss mouse	5	Distilled water	NM			**IM:** ↑ antibody and cell-mediated immune response (total white blood cell, neutrophil and lymphocyte counts) as adjuvant to hepatitis B vaccine.	PC (in vivo)	Yes	No	(Onah et al., 2019)
10	Aqueous extract	Leaf	200 mg/kg to 800 mg/kg	Wistar rat	5	Untreated group	NM			**IS:** ↑ CD4+ count.	PC (in vivo)	No	Yes	(Momoh et al., 2010)
11	80% ethanol extract	Leaf	200 mg/kg	Wistar rat	6	Saline (non-diabetic group)	NM			**IM:** ↓ CD4+ count.	PC (in vivo)	Yes	No	(Eyong et al., 2011)
12	Ethanol extract	Leaf	UC	Swiss rat	8	Normal saline	NM			No significant changes in white cell counts.	PC (in vivo)	No	No	(Kola, 2007)

No.	Formulation	PP	Dose	Methodology				Antiviral		Anti-inflammatory (AI) / Immunomodulatory (IM)/ Immunostimulant (IS)		Quality		Ref.
				Study model	Sample size (per group	Comparator	Ethics approval	Outcome	Study type	Outcome	Study type	PI	PA	
13	Aqueous extract	Leaf	UC (Two handfuls in 200 mL water, no quantitative analysis)	HIV-infected patients who took the first line antiretroviral drugs	40	Antiretroviral drugs	NM			**IM:** ↑ CD4+ count when given in combination with multivitamin (compared to either multivitamin or herb alone)	C (RCT)	No	No	(Momoh et al., 2012)
**B.** ***Azadirachta indica*** **A. Juss.**														
1	NA	NA	NA	Molecular modelling	NA	NA	NA	No effects on Hepatitis C virus NS3/4A protease	PC (in silico)			No	No	(Ashfaq et al., 2016)
2	Isolated Chemical sulfated derivatives (P1S and P2S)	Leaf	25 µg/mL to 200 µg/mL	HEp-2 cells	NA	Human alfa-2B interferon	NA	No effects. on Polio virus type1	PC (in vitro)			Yes	No	(Faccin-Galhardi et al., 2012)
3	Aqueous and ethanolic extract	Seed	0.38096 g/kg	Bal/b C mouse	20	Extract-prednisolone treated group	NM			**IM:** ↑ interferon-γ levels and titer of anti-Brucella antibodies	PC (in vivo)	No	No	(Faal, 2012)

No.	Formulation	PP	Dose	Methodology				Antiviral		Anti-inflammatory (AI) / Immunomodulatory (IM)/ Immunostimulant (IS)		Quality		Ref.
				Study model	Sample size (per group	Comparator	Ethics approval	Outcome	Study type	Outcome	Study type	PI	PA	
4	Aqueous extract	UC	UC	Human pathogenic bacteria *Staphylococcus aureus*	NA	Untreated bacteria	NA			**IM:** Phagocytic activity against *Staphylococcus aureus*	PC (in vitro)	No	No	(Saeed et al., 2017)
5	Aqueous extract	Leaf	8.8 mg/kg to 26.7 mg/kg	Swiss Wistar mouse	10	Phosphate buffer saline	UC			**IM:** ↑ IL-2 and T-lymphocytes	PC (in vivo)	Yes	No	(Venugopalan et al., 2011)
6	NA	NA	NA	Molecular modelling	NA	Quercetin	NA	In vitro: No effects on dengue virus<br><br>In silico: Kaempferol-3-*O*-rutinoside, rutin, hyperoside, epicatechin (predicted negative binding affinity as better viral	PC (in silico)			No	No	(Dwivedi et al., 2020)

No.	Formulation	PP	Dose	Methodology				Antiviral		Anti-inflammatory (AI) / Immunomodulatory (IM)/ Immunostimulant (IS)		Quality		Ref.
				Study model	Sample size (per group	Comparator	Ethics approval	Outcome	Study type	Outcome	Study type	PI	PA	
6								protease inhibitors compared to reference compound)						
7	Gelation capsule containing acetone-water extract of *A. indica*	Leaf	1,000 mg of daily for 30 days	HIV patients	10	Before-after	Yes	No effects on HIV.	C (quasi-experimental)			No	No	(Udeinya et al., 2004)
**C. *Nigella sativa* L.**														
1	Soft gelatin capsule containing 500 mg of dried ethanol (96%) extract of *N. sativa*	Seed	500 mg twice daily	Hepatitis C patients	60	Untreated group	Yes	↓ Hepatitis C viral load, alpha fetoprotein, abnormal liver enzymes.	C (single-arm pilot)			No	No	(Abdel-Moneim et al., 2013)
2	Concoction of *N. sativa*	NM	10 mL twice daily for 6 months	HIV male patient	1	NA	NA	↓ CD4 count, HIV viral load, signs & symptoms associated with HIV.	C (case report)			No	No	(Onifade et al., 2013)

No.	Formulation	PP	Dose	Methodology				Antiviral		Anti-inflammatory (AI) / Immunomodulatory (IM)/ Immunostimulant (IS)		Quality		Ref.
				Study model	Sample size (per group	Comparator	Ethics approval	Outcome	Study type	Outcome	Study type	PI	PA	
3	Mixture of dried powder of N. sativa and liquid honey (60: 40 respectively)	Seed	10 mL thrice daily for a year	HIV female patient	1	NA	NA	↓ HIV viral load.<br>↑ CD4 count.	C (case report)			No	No	(Onifade et al., 2015)
4	Oil of *N. sativa*	Seed	5 mg/kg	Bal/b C mouse	5	Phosphate buffer saline	NM	↓ murine cytomegalo virus load.	PC (in vivo)	**IM:**<br>↑ CD4, CD3, IFN-γ, macrophage levels.	PC (in vivo)	No*	No*	(Salem et al., 2000)
5	Ethanol extract	Seed	1/50 diluted concentration	HeLa cells	NA	Uninfected cells	NA	↓ murine coronavirus mouse hepatitis virus strain A59 load.	PC (in vitro)	**AI:**<br>↑ IL-8, TRPA1, TRPC4, TRPM6, TRPM7, TRPM8, TRPV4 expression levels	PC (in vitro)	No	No	(Ulasli et al., 2014)
6	90% ethanol extract	Seed	0.2 mg / 0.2 mL	Chicken embryonated egg	12	Untreated control	NA			**IM:**<br>↑ aggregation of lymphocytes, lymphoblasts and macrophages	PC (in ovo)	No	No	(Khan et al., 2018)

No.	Formulation	PP	Dose	Methodology				Antiviral		Anti-inflammatory (AI) / Immunomodulatory (IM)/ Immunostimulant (IS)		Quality		Ref.
				Study model	Sample size (per group	Comparator	Ethics approval	Outcome	Study type	Outcome	Study type	PI	PA	
**D. *Eurycoma longifolia* Jack**														
1	Hard gelatin capsule containing 200 mg of standardised aqueous extract of *E. longifolia* and 30 mg of fatty acid sucrose esters	Root	200 mg daily for 4 weeks	Middle aged female and male subjects	84	Rice powder (placebo)	Yes			**IS:** ↑ scoring of immunological vigor, immunological grade, number of total, naïve, CD4+ T cells	C (RCT)	No*	Yes *	(George et al., 2016)
2	Capsule containing 75 mg of standardised aqueous extract	Root	150 mg daily for 7 days	Healthy athletes	9	Flour (placebo)	Yes			**IS:** ↑ NK cell counts	C (RCT)	No	No	(Muhamad et al., 2015)
3	Purified polysaccharide fraction	Root	125 µg/mL to 2,000 µg/mL	RAW264.7 cells	NA	Untreated cells	NA			**IM:** ↑ phagocytic ability, NO, TNF-α, IL-6 secretion	PC (in vitro)	Yes	Yes	(He et al., 2019)

No.	Formulation	PP	Dose	Methodology				Antiviral		Anti-inflammatory (AI) / Immunomodulatory (IM)/ Immunostimulant (IS)		Quality		Ref.
				Study model	Sample size (per group	Comparator	Ethics approval	Outcome	Study type	Outcome	Study type	PI	PA	
4	Isolated compounds (quassinoids, alkaloids, chromone derivative, triterpenoid, flavonoid) from methanol extract	Root	0.5 µmol/L and 0.7 µmol/L	HEK293/NF-κB-luc cells	NA	Parthenolide	NA			**AI:** Inhibited NF-κB	PC (in vitro)	Yes	Yes	(Tran et al., 2014)
5	Isolated alkaloid (9-methoxycanthin-6-one) isolated from hairy roots from hairy root culture	Root	3.5 µmol/L to 11.6 µmol/L	RAW264.7 cells Murine peritoneal macrophages Human THP-1 macrophages	NA	Lipopolysaccharide	NA			**AI:** Inhibited IL-6, TNF-α	PC (in vitro)	Yes	Yes	(Tran et al., 2018)

No.	Formulation	PP	Dose	Methodology				Antiviral		Anti-inflammatory (AI) / Immunomodulatory (IM)/ Immunostimulant (IS)		Quality		Ref.
				Study model	Sample size (per group	Comparator	Ethics approval	Outcome	Study type	Outcome	Study type	PI	PA	
6	Isolated phenolic compounds (piscidinol A, 24-epi-piscidinol A, bourjotinolone A, scopoletin) from 70% ethanol extract	Root	40 µmol/L	RAW264.7 cells	NA	Lipopolysaccharide	NA			**AI:** Inhibited iNOS, IL-6, NF-κB	PC (in vitro)	Yes	Yes	(Ruan et al., 2019)
7	Alkaloid fraction from methanol extract	Root	28.18 µg/mL to 150 µg/mL	RAW264.7 cells	NA	Alpha-tubulin Cardamonin	NA			**AI:** Inhibited NO, iNOS, COX-2	PC (in vitro)	Yes	Yes	(Hien et al., 2019)
8	Isolated alkaloid (7-methoxy-(9H-b-carbolin-1-il)-(E)-1-propenoic acid) from hairy root culture	Root	In vitro: 3 µg/mL to 30 µg/mL<br><br>In vivo: 30 mg/kg	RAW264.7 cells	NA	Lipopolysaccharide	NA			**AI:** Inhibited NO, PGE2, IL-6	PC (in vitro)	No	No	(Nguyen et al., 2016)

No.	Formulation	PP	Dose	Methodology				Antiviral		Anti-inflammatory (AI) / Immunomodulatory (IM)/ Immunostimulant (IS)		Quality		Ref.
				Study model	Sample size (per group	Comparator	Ethics approval	Outcome	Study type	Outcome	Study type	PI	PA	
9	Standardised aqueous extract	Root	In vitro: 33.84 µg/L to 119 µg/L<br><br>In vivo: 100 mg/kg twice daily	AG129 mouse	6	Phosphate buffer saline	Yes	In vitro: ↓ viral yield<br><br>In vivo: ↓ body weight, ↓ viral load, ↑ platelet counts in dengue virus infected mice	PC (in vitro, in vivo)			No*	No*	(George et al., 2019)

Table S1b. Safety

No.	Formulation	PP	Dose	Methodology					Safety		Quality		Ref.
				Study model	Sample size (per group)	Comparator	Guideline used	Ethics approval	Outcome	Study Type	PI	PA	
**A. *Gymnanthemum amygdalinum* (Delile) Sch. Bip.**													
1	Acetone extract	Leaf	100 mg/kg to 200 mg/kg	Mice	5	Normal saline	NM	NM	No acute toxicity.	PC (in vivo)	Yes	UC	(Adedapo et al., 2014)
2	Aqueous extract	Leaf	250 mg/kg to 1,000 mg/kg	Young female Swiss albino mice	3	Distilled water	NM	NM	Dose dependent weight changes. No acute toxicity and mortality. No changes in organ weight	PC (in vivo)	Yes	No	(Onah et al., 2019)
3	Aqueous extract	Leaf	5,000 mg/kg	Male albino rats	8	NM	NM	NM	No acute toxicity and mortality	PC (in vivo)	Yes	No	(Oguwike et al., 2013)
4	Aqueous extract	Leaf	5,000 mg/kg	Nulliparous and non-pregnant female Sprague Dawley rats	1	NM	OECD 425	Yes	No acute toxicity and mortality	PC (in vivo)	Yes	No	(Zakaria et al., 2016)
5	Aqueous extract	Leaf	200 mg/kg to 600 mg/kg	Wistar white albino rats	10	Goodwin's physiological solution	OECD test guidelines	NM	Kidney congestion after 28 days administration	PC (in vivo)	Yes	No	(Nabukenya et al., 2014).
6	Ethanol extract	Leaf	100 mg/kg to 600 mg/kg	Male Sprague-Dawley rats	10	Distilled water	NM	Yes	Testicular toxicity at higher doses (300 and 600 mg/kg)	PC (in vivo)	Yes	No	(Saalu et al., 2013)

No.	Formulation	PP	Dose	Methodology					Safety	Quality			Ref.
				Study model	Sample size (per group)	Comparator	Guideline used	Ethics approval	Outcome	Study Type	PI	PA	
**B. *Azadirachta indica* A. Juss.**													
1	Aqueous extract	Leaf	8.8 mg/kg to 26.7 mg/kg	Swiss Wistar mice	6	NM	OECD 423	UC	No acute toxicity. No histopathological changes in liver or renal	PC (in vivo)	Yes	No	(Venugopalan et al., 2011)
2	In vitro (potential bioflavanoids): 1) kaempferol-3-O-β-rutinoside (0.01-100 µM) 2) epicatechin (0.001-1 mM)<br><br>In silico: Bioflavanoids	NA	In vitro: UC	Baby hamster kidney cell-21	NA	Quercetin	NA	NA	No significant cell toxicity	PC (in silico and in vitro)	No	No	(Dwivedi et al., 2020)
3	Acetone-water extract	Leaf	UC	Ambulatory HIV-positive volunteers	10	NA	NA	Yes	No adverse/side effects related to the extract during the treatment and follow-up period	C (Quasi)	No	No	(Udeinya et al., 2004)

No.	Formulation	PP	Dose	Methodology					Safety	Quality			Ref.
				Study model	Sample size (per group)	Comparator	Guideline used	Ethics approval	Outcome	Study Type	PI	PA	
**C. *Nigella sativa* L.**													
1	Ethanol extract	Seed	1/50 diluted concentration	HeLa-epithelial carcinoembryonic antigen-related cell adhesion molecule 1a)	NA	NA	NA	NA	Cytotoxicity: Toxicity resolved after 24 hr	PC (in vitro)	No	No	(Ulasli et al., 2014)
2	Thymoquinone	NA	6 µmol/L to 25 µmol/L	EBV-infected B cell lines	NA	NA	NA	NA	Cytotoxicity: Reduced survivability and growth of Epstein Barr Virus (EBV)-infected B cell lines, including inhibited EBV gene expression. Low toxicity effect on normal human PBMC and PLF cells	PC (in vitro)	NA	NA	(Zihlif et al., 2013)
3	Capsule containing 750 mg of N. sativa	NM	1,500 mg twice daily for 3 months	30-45 year old men	19-20	NA	NM	NM	Reduced body weight, waist circumference, systolic blood pressure. Improved clinical symptoms among central obesity in men	C (RCT)	No	No	(Datau et al., 2010)

No.	Formulation	PP	Dose	Methodology					Safety		Quality		Ref.
				Study model	Sample size	Comparator	Guideline used	Ethics approval	Outcome	Study Type	PI	PA	
4	Capsule containing 100 - 200 mg of standardised aqueous extract of *N. sativa*	Seed	200 mg to 400 mg for 8 weeks	Healthy male volunteers	33-39	NA	NM	Yes	No observable complications in patients with mild hypertension	C (RCT)	Yes	Yes	(Dehkordi et al., 2008)
5	Capsule containing 500 mg powdered *N. sativa*	Seed	1,000mg daily for 9 weeks	Healthy elderly male volunteers	20	NA	NM	UC	No significant changes in biochemical markers of cardiac, liver, kidney function on healthy elderly volunteers	C (RCT)	Yes	No	(Bin Sayeed et al., 2013)
6	Capsule containing 500 mg crushed powdered *N. sativa*	Seed	1,000 mg twice daily for 6 weeks	Adult men and women of Pakistani origin	59-64	NA	NM	Yes	No effect on lipid levels, blood sugar, blood pressure, and body weight in hypercholesterolaemic patients	C (RCT)	No	No	(Qidwai et al., 2009)
7	Mixture of *N. sativa* oil, honey and water (5:5:5)	Seed	15 mL once daily for 8 weeks	Patients diagnosed as with functional dyspepsia	35	NA	NM	Yes	Caused mild gastrointestinal effects (nausea, bloating, and burning sensation) on patients with functional dyspepsia	C (RCT)	No	No	(Mohtashami et al., 2015)

No.	Formulation	PP	Dose	Methodology					Safety		Quality		Ref.
				Study model	Sample size	Comparator	Guideline used	Ethics approval	Outcome	Study Type	PI	PA	
8	*N. sativa oil*	Seed	2.5 mL twice daily for 2 months	Type II diabetic patients	35	NA	NM	Yes	Caused mild gastrointestinal effects (nausea) on diabetic patients	C (RCT)	No	No	(Hosseini et al., 2013).
9	*N. sativa oil*	Seed	2.5 mL twice daily for 2 months	Healthy volunteers	35	NA	NM	Yes	Caused mild gastrointestinal effects (nausea) on healthy volunteers	C (RCT)	No	No	(Fallah Huseini et al., 2013)
10	Soft gelatin capsule containing 450 mg of *N. sativa* oil	Seed	450 mg capsule thrice daily for 3 months	Patients with hepatitis C virus (HCV) infection	30	NA	NM	Yes	Caused epigastric pain and hypoglycemia among Hepatitis C patients	C (single-arm pilot)	No	No	(Barakat et al., 2013)
11	96% ethanol extract	Seed	2.5 g twice daily for 7 days	Healthy volunteers	4	NA	NM	Yes	Inhibited activities of CYP3A4 and CYP2D6	C (pilot)	No	No	(Al-Jenoobi et al., 2010)
12	Hexane extract	Seed	Acute (single dose)<br>Oral: 5 - 50 mL/kg<br>i.p.: 0.25 - 4.0 mL/kg<br><br>Chronic<br>Oral: 2 mL/kg once daily for 12 weeks	Iops ofa mice	Acute: 1<br><br>Chronic: 12	Distilled water	NM	NM	Acute<br>Oral: LD50 = 28.8 mL/kg<br>i.p.: LD50 = 2.06 mL/kg<br><br>Chronic<br>No effect on liver enzymes	PC (in vivo)	No	No	(Zaoui et al., 2002)

No.	Formulation	PP	Dose	Methodology					Safety		Quality		Ref.
				Study model	Sample size	Comparator	Guideline used	Ethics approval	Outcome	Study Type	PI	PA	
13	Dried powder mixed into rat chow pellet	Seed	1.0 g/kg daily for 28 days	Male Sprague Dawley rats	6	Normal diet	NM	NM	No effect on liver enzymes	PC (in vivo)	Yes	No	(Dollah et al., 2013)
14	Aqueous extract	Seed	6 g/kg	Male mice	8	Distilled water, sesame oil	NM	NM	Observable degenerative changes on liver	PC (in vivo)	No	No	(Vahdati - Mashhadian et al., 2005)
15	Aqueous extract	Seed	6.4 g/kg to 60 g/kg	Mus musculus mice	5	Distilled water	UC	NM	Mortality was observed after 2 to 5 weeks of treatment with 6.4, 21, 60 g/kg doses<br>Observable hepatoxicity effect at dose of 21 and 60 g/kg	PC (in vivo)	Yes	No	(Bensiameur-Touati et al., 2017)
16	Thymoquinone	NA	Acute (single dose)<br>2000 and 3000 g/kg<br><br>Subchronic (90 days)<br>30 mg/kg to 90 mg/kg	Male Swiss albino mice	8	Corn oil	NM	NM	Acute<br>LD50 = 2.4 g/kg<br>Some animals showed hypoactivity and marked difficulty in respiration before death within the first 3 hr of dosing<br>Reduced GSH level	PC (in vivo)	NA	NA	(Badary et al., 1998)

No.	Formulation	PP	Dose	Methodology					Safety		Quality		Ref
				Study model	Sample size	Comparator	Guideline used	Ethics approval	Outcome	Study Type	PI	PA	
16									in the liver, kidneys, heart.<br>Increased level of plasma urea, creatinine, ALT, LDH, CPK<br><br>Subchronic<br>No observable toxicity effect, except increased fasting plasma glucose level				
17	Thymoquinone	NA	i.p.: 20 mg/kg to 40 mg/kg<br><br>Oral: 200 mg/kg to 500 mg/kg	Male and female Wistar rats	8	0.5% DMSO, olive oil	NM	UC	i.p.<br>Showed signs of toxicity related to acute pancreatitis<br><br>Oral<br>Showed transient toxicity signs.<br>Bowel obstruction complications lead to 2 deaths among the 8 animals at dose 500 mg/kg	PC (in vivo)	NA	NA	(Abukhader, 2012)

No.	Formulation	PP	Dose	Methodology					Safety		Quality		Ref
				Study model	Sample size	Comparator	Guideline used	Ethics approval	Outcome	Study Type	PI	PA	
**D. *Eurycoma longifolia* Jack**													
1	Purified polysaccharide fraction	Root	125 µg/mL to 2,000 µg/mL	RAW264.7 cells	NA	NA	NA	NA	Cytotoxicity: No toxic effect on RAW264.7 cells	PC (in vitro)	Yes	Yes	(He et al., 2019)
2	Isolated alkaloid (7-methoxy-(9H-b-carbolin-1-il)-(E)-1-propenoic acid) from hairy root culture	Root	In vitro: 3 µg/mL to 30 µg/mL<br><br>In vivo: 30 mg/kg	Male C57BL/6 mice	8	NA	NA	Yes	Increased the survival rate of C57BL/6 mice	PC (in vivo)	No	No	(Nguyen et al., 2016)
3	Standardised aqueous extract	Root	In vitro: 33.84 µg/L to 119 µg/L	Vero cell	NA	NA	NA	NA	Cytotoxicity: No observable toxic effect on Vero cells	PC (in vitro)	No*	No*	(George et al., 2019)
4	Powder resuspended in sterile water	Root	In vitro: 0.3125 mg/plate to 5 mg/plate<br><br>In vivo: Ames test: 0.6 g/kg to 2 g/kg	Male and female Wistar rats	Acute: 8<br><br>Sub-acute: 10	Sterile water	OECD 407 and 408	NM	In vitro: No observable mutagenic or clastogenic effect<br><br>In vivo: Ames test: No observable mutagenic effect	PC (in vitro, in vivo)	No	No	(Li et al., 2013)

No.	Formulation	PP	Dose	Methodology					Safety		Quality		Ref
				Study model	Sample size	Comparator	Guideline used	Ethics approval	Outcome	Study Type	PI	PA	
4			Acute toxicity: 1 g/kg to 6 g/kg<br>Subacute (4 weeks), subchronic (13 weeks) toxicity: 0.6 g/kg to 2 g/kg		Sub-chronic: 10 males and 20 females				Acute: LD_{50} > 6 g/kg<br>Subacute, subchronic: Observable changes in few serum biochemical parameters				
5	Methanol: chloroform (1:1) extract	Root	0.005 mL	Two human cell lines, Hep2 and HFL1	NA	NA	NA	NA	Mutagenicity: No observable toxic effect	PC (in vitro)	Yes	No	(Mohd-Fuat et al., 2007)
6	Methanol extract	Root	Acute toxicity: 2,000 mg/kg<br><br>Reproductive toxicity, teratogenicity: 10 mg/kg to 100 mg/kg	Sprague–Dawley rats	Acute: 10<br><br>Reproductive: 30	Distilled water	OECD 425	Yes	Acute: LD_{50} for female rats = 1293 mg/kg, LD_{50} for male rats > 2,000 mg/kg.<br><br>Reproductive, teratogenicity: NOAEL = 100 mg/kg	PC (in vivo)	Yes	Yes	(Low et al., 2014)
7	Aqueous extract	Root	250 mg/kg to 1,000 mg/kg once daily for 5 weeks	Male Sprague Dawley rats	8	Distilled water	NM	NM	No observable toxic effect on pancreas.	PC (in vivo)	No	No	(Hamoud et al., 2013)

No.	Formulation	PP	Dose	Methodology					Safety		Quality		Ref.
				Study model	Sample size	Comparator	Guideline used	Ethics approval	Outcome	Study Type	PI	PA	
8	Standardised aqueous extract	Root	Acute toxicity: 5,000 mg/kg<br><br>Subacute toxicity (28 days): 600 mg/kg to 2,400 mg/kg	Male Sprague Dawley rats	Acute: 5<br><br>Subacute: 8	NM	OECD 407	Yes	Acute: LD_{50} = 5,000 mg/kg<br><br>Subacute: Observable hepatoxic effect	PC (in vivo)	No*	No*	(Shuid et al., 2011)
9	Capsule containing 100 mg of a proprietary standardised aqueous extract	Root	200 mg capsules twice daily for 9 months	Patients suffering from late-onset hypogonadism	76	NA	NM	Yes	Improved semen & sperm quality in patients.	C	No*	No*	(Tambi et al., 2010)
10	Dried powder of standardised aqueous extract	Root	In vitro: 5 mg/plate<br><br>In vivo: 100 mg/kg to 500 mg/kg	Male and female NMRI mice	10	Cyclophosphamide	OECD 474	UC	No observable mutagenic or clastogenic effect	PC (in vitro, in vivo)	No*	No*	(Yee et al., 2014)

* Marketed and registered natural products in Malaysia

Table header abbreviations: AI = Anti-inflammatory; IM = Immunomodulatory; IS = Immunostimulant; PI = Plant Identification; PA = Plant authentication (via chemical fingerprinting/ qualitative assessment of markers); PP= Plant Part

Table content abbreviations: C= Clinical; DD= Dose dependent; NA = Not applicable (i.e., data not applicable due to study methodology/type); NM = Not mentioned (i.e., data not reported); PC = Preclinical; RCT = Randomised controlled trial; UC = Unclear (i.e., ambiguity in data reported)

References:

1. Abdel-Moneim, A., Morsy, B.M., Mahmoud, A.M., Abo-Seif, M.A., and Zanaty, M.I. (2013). Beneficial therapeutic effects of *Nigella sativa* and/or *Zingiber officinale* in HCV patients in Egypt. EXCLI journal. 12, 943-955.
2. Abukhader, M.M. (2012). The effect of route of administration in thymoquinone toxicity in male and female rats. Indian J Pharm Sci. 74(3), 195-200. doi: 10.4103/0250-474X.106060.
3. Adedapo, A.A., Aremu, O.J., and Oyagbemi, A.A. (2014). Anti-oxidant, anti-inflammatory and antinociceptive properties of the acetone leaf extract of *Vernonia amygdalina* in some laboratory animals. Adv Pharm Bull. 4(Suppl 2), 591-598. doi: 10.5681/apb.2014.087.
4. Al-Jenoobi, F.I., Al-Thukair, A.A., Abbas, F.A., Ansari, M.J., Alkharfy, K.M., Al-Mohizea, A.M., et al. (2010). Effect of black seed on dextromethorphan O- and N-demethylation in human liver microsomes and healthy human subjects. Drug Metab Lett. 4(1), 51-55. doi: 10.2174/187231210790980435.
5. Asante, D.-B., Henneh, I.T., Acheampong, D.O., Kyei, F., Adokoh, C.K., Ofori, E.G., et al. (2019). Anti-inflammatory, anti-nociceptive and antipyretic activity of young and old leaves of *Vernonia amygdalina*. Biomed Pharmacother. 111, 1187-1203. doi: 10.1016/j.biopha.2018.12.147.
6. Ashfaq, U.A., Jalil, A., and ul Qamar, M.T. (2016). Antiviral phytochemicals identification from *Azadirachta indica* leaves against HCV NS3 protease: an in silico approach. Nat Prod Res. 30(16), 1866-1869.
7. Badary, O.A., Al-Shabanah, O.A., Nagi, M.N., Al-Bekairi, A.M., and Elmazar, M.M.A. (1998). Acute and subchronic toxicity of thymoquinone in mice. Drug Dev Res. 44(2-3), 56-61. doi: 10.1002/(SICI)1098-2299(199806/07)44:2/3<56::AID-DDR2>3.0.CO;2-9.
8. Barakat, E.M.F., El Wakeel, L.M., and Hagag, R.S. (2013). Effects of *Nigella sativa* on outcome of hepatitis C in Egypt. World J Gastroenterol. 19(16), 2529-2536. doi: 10.3748/wjg.v19.i16.2529.
9. Bensiameur-Touati, K., Kacimi, G., Haffaf, E.M., Berdja, S., and Aouichat-Bouguerra, S. (2017). In vivo subacute toxicity and antidiabetic effect of aqueous extract of *Nigella sativa*. Evid Based Complement Alternat Med. 2017, 8427034. doi: 10.1155/2017/8427034.
10. Bin Sayeed, M.S., Asaduzzaman, M., Morshed, H., Hossain, M.M., Kadir, M.F., and Rahman, M.R. (2013). The effect of *Nigella sativa* Linn. seed on memory, attention and cognition in healthy human volunteers. J Ethnopharmacol. 148(3), 780-786. doi: 10.1016/j.jep.2013.05.004.
11. Datau, E.A., Wardhana, Surachmanto, E.E., Pandelaki, K., Langi, J.A., and Fias (2010). Efficacy of *Nigella sativa* on serum free testosterone and metabolic disturbances in central obese male. Acta Med Indones. 42(3), 130-134.
12. Dehkordi, F.R., and Kamkhah, A.F. (2008). Antihypertensive effect of *Nigella sativa* seed extract in patients with mild hypertension. Fundam Clin Pharmacol. 22(4), 447-452. doi: 10.1111/j.1472-8206.2008.00607.x.

13. Dollah, M.A., Parhizkar, S., Latiff, L.A., and Bin Hassan, M.H. (2013). Toxicity effect of *Nigella sativa* on the liver function of rats. Adv Pharm Bull. 3(1), 97-102. doi: 10.5681/apb.2013.016.
14. Dwivedi, V.D., Bharadwaj, S., Afroz, S., Khan, N., Ansari, M.A., Yadava, U., et al. (2020). Anti-dengue infectivity evaluation of bioflavonoid from *Azadirachta indica* by dengue virus serine protease inhibition. J Biomol Struct Dyn. 1-14. doi: 10.1080/07391102.2020.1734485.
15. Eyong, E.U., Atangwho, I.J., David-Oku, E., Agiang, M.A., and Ebong, P.E. (2011). Haematological and immunological effect of coadministration of extracts of *Vernonia amygdalina* and *Azadirachta indica* on normal and diabetic rats. Afr J Biotechnol. 10(50), 10258-10262.
16. Faal, T.J. (2012). The immunomodulatory effect of Neem (*Azadirachtaindica*) seed aqueous, ethanolic extracts and *Candida albicans* cell wall mannoproteins on immune response in mice vaccinated with Brucella Rev-1. Iraqi .J Vet Med 36(1), 120-127.
17. Faccin-Galhardi, L.C., Yamamoto, K.A., Ray, S., Ray, B., Linhares, R.E.C., and Nozawa, C. (2012). The in vitro antiviral property of *Azadirachta indica* polysaccharides for poliovirus. J Ethnopharmacol. 142(1), 86-90.
18. Fallah Huseini, H., Amini, M., Mohtashami, R., Ghamarchehre, M.E., Sadeqhi, Z., Kianbakht, S., et al. (2013). Blood pressure lowering effect of *Nigella sativa* L. seed oil in healthy volunteers: a randomized, double-blind, placebo-controlled clinical trial. Phytother Res. 27(12), 1849-1853. doi: 10.1002/ptr.4944.
19. George, A., Suzuki, N., Abas, A.B., Mohri, K., Utsuyama, M., Hirokawa, K., et al. (2016). Immunomodulation in middle-aged humans via the ingestion of Physta® standardized root water Extract of *Eurycoma longifolia* Jack--A randomized, double-blind, placebo-controlled, parallel study. Phytother Res. 30(4), 627-635. doi: 10.1002/ptr.5571.
20. George, A., Zandi, K., Biggins, J., Chinnappan, S., Hassandarvish, P., and Yusof, A. (2019). Antiviral activity of a standardized root water extract of *Eurycoma longifolia* (Physta®) against dengue virus. Trop Biomed. 36(2), 412-421.
21. Hamoud, H., and Qamar, U. (2013). Effect of long-term use of *Eurycoma longifolia* Jack on the pancreas in rats: histological assessment. Regen Res. 2(2), 22-25.
22. He, P., Dong, Z., Wang, Q., Zhan, Q.-P., Zhang, M.-M., and Wu, H. (2019). Structural characterization and immunomodulatory activity of a polysaccharide from *Eurycoma longifolia*. J Nat Prod. 82(2), 169-176. doi: 10.1021/acs.jnatprod.8b00238.
23. Hien, D., Long, T., Thao, T., Lee, J.-H., Trang, D., Minh, N., et al. (2019). Anti-inflammatory effects of alkaloid enriched extract from roots of *Eurycoma longifolia* Jack. Asian Pac J Trop Biomed. 9(1), 18-23. doi: 10.4103/2221-1691.250265.
24. Hosseini, M.S., Mirkarimi, S.A., Amini, M., Mohtashami, R., Kianbakht, S., and Fallah Huseini, H. (2013). Effects of *Nigella sativa* L. seed oil in type II diabetic patients: a randomized, double-blind, placebo- controlled clinical trial. J Med Plants. 12(47), 93-99.
25. Im, E., Ae, A., Bn, U., and Po, U. (2016). Immuno-modulatory properties of prebiotics extracted from *Vernonia amygdalina*. Afr J Tradit Complement Altern Med. 13(6), 11-17. doi: 10.21010/ajtcam.v13i6.3.

26. Khan, A., Tipu, Y., Shafee, M., Khan, N., Masood, M., Kiani, T., et al. (2018). In-ovo antiviral effect of *Nigella sativa* extract against Newcastle disease virus in experimentally infected chicken embryonated eggs. Pak Vet J. 2074-7764. doi: 10.29261/pakvetj/2018.075.
27. Kola, O. (2007). Anti-inflammatory activity of ethanolic leaf extract from *Vernonia amygdalina* on the immune system of Swiss Albino rats dosed with Clostridium sporogenes (NC13532). Res J Med Sci. 1(2), 127-131.
28. Li, C.-H., Liao, J.-W., Liao, P.-L., Huang, W.-K., Tse, L.-S., Lin, C.-H., et al. (2013). Evaluation of acute 13-Week subchronic toxicity and genotoxicity of the powdered root of Tongkat Ali (*Eurycoma longifolia* Jack). Evid Based Complement Alternat Med. 2013, 102987-102987. doi: 10.1155/2013/102987.
29. Low, B.S., Das, P.K., and Chan, K.L. (2014). Acute, reproductive toxicity and two-generation teratology studies of a standardized quassinoid-rich extract of *Eurycoma longifolia* Jack in Sprague-Dawley rats. Phytother Res. 28(7), 1022-1029. doi: 10.1002/ptr.5094.
30. Madzuki, I.N., Lau, S.F., Abdullah, R., Mohd Ishak, N.I., and Mohamed, S. (2019). *Vernonia amygdalina* inhibited osteoarthritis development by anti-inflammatory and anticollagenase pathways in cartilage explant and osteoarthritis-induced rat model. Phytother Res. 33(7), 1784-1793. doi: 10.1002/ptr.6366.
31. Mohd-Fuat, A.R., Kofi, E.A., and Allan, G.G. (2007). Mutagenic and cytotoxic properties of three herbal plants from Southeast Asia. Trop Biomed. 24(2), 49-59.
32. Mohtashami, R., Huseini, H.F., Heydari, M., Amini, M., Sadeqhi, Z., Ghaznavi, H., et al. (2015). Efficacy and safety of honey based formulation of *Nigella sativa* seed oil in functional dyspepsia: A double blind randomized controlled clinical trial. J Ethnopharmacol. 175, 147-152. doi: 10.1016/j.jep.2015.09.022.
33. Momoh, M., Adikwu, M., and Oyi, A. (2010). *Vernonia amygdalina* extract and CD4+ cell counts: an immune study. GJBB. 5(2), 92-96.
34. Momoh, M.A., Muhamed, U., Agboke, A.A., Akpabio, E.I., and Osonwa, U.E. (2012). Immunological effect of aqueous extract of *Vernonia amygdalina* and a known immune booster called immunace(®) and their admixtures on HIV/AIDS clients: a comparative study. Asian Pac J Trop Biomed. 2(3), 181-184. doi: 10.1016/S2221-1691(12)60038-0.
35. Muhamad, A., Ooi, F.K., and Chen, C.K. (2015). Effects of *Eurycoma longifolia* on natural killer cells and endurance running performance. Int J Sports Sci. 5, 93-98.
36. Nabukenya, I., Rubaire-Akiiki, C., Mugizi, D., Kateregga, J., and Olila, D. (2014). Sub-acute toxicity of aqueous extracts of *Tephrosia vogelii, Vernonia amygdalina* and *Senna occidentalis* in rats. Nat Prod Chem Res. 2(5), 1000143. doi: 10.4172/2329-6836.1000143.
37. Nguyen, H.D., Choo, Y.Y., Nguyen, T.D., Nguyen, H.N., Chau, V.M., and Lee, J.H. (2016). 7-methoxy-(9H-β-carbolin-1-il)-(E)-1-propenoic acid, a β-carboline alkaloid from *Eurycoma longifolia*, exhibits panti-inflammatory effects by activating the nrf2/heme oxygenase-1 Pathway. J Cell Biochem. 117(3), 659-670. doi: 10.1002/jcb.25315.
38. Oguwike, F., Offor, C., Onubeze, D., and Nwadioha, A. (2013). Evaluation of activities of bitterleaf (*Vernonia amygdalina*) extract on haemostatic and biochemical profile of induced male diabetic albino rats. J Med Dent Sci. 11(2), 60-64.

39. Omoregie, E.S., and Pal, A. (2016). Antiplasmodial, antioxidant and immunomodulatory activities of ethanol extract of *Vernonia amygdalina* del. Leaf in Swiss mice. Avicenna J Phytomedicine. 6(2), 236-247.
40. Onah, I.A., Onuigbo, E.B., and Odimegwu, D.C. (2019). Adjuvant effect of *Vernonia amygdalina* leaf extract on host immune response to hepatitis B virus subunit vaccine. Pharmazie. 74(3), 179-185. doi: 10.1691/ph.2019.8920.
41. Onasanwo, S.A., Oyebanjo, O.T., Ajayi, A.M., and Olubori, M.A. (2017). Anti-nociceptive and anti-inflammatory potentials of *Vernonia amygdalina* leaf extract via reductions of leucocyte migration and lipid peroxidation. J Intercult Ethnopharmacol. 6(2), 192-198. doi: 10.5455/jice.20170330010610.
42. Onifade, A.A., Jewell, A., and Okesina, A. (2015). Seronegative conversion of an HIV positive subject treated with *Nigella sativa* and honey. AJID. 9(2), 47-50.
43. Onifade, A.A., Jewell, A.P., and Adedeji, W.A. (2013). *Nigella sativa* concoction induced sustained seroreversion in HIV patient. Afr J Tradit Complement Altern Med. 10(5), 332-335.
44. Qidwai, W., Hamza, H.B., Qureshi, R., and Gilani, A. (2009). Effectiveness, safety, and tolerability of powdered *Nigella sativa* (kalonji) seed in capsules on serum lipid levels, blood sugar, blood pressure, and body weight in adults: results of a randomized, double-blind controlled trial. J Altern Complement Med. 15(6), 639-644. doi: 10.1089/acm.2008.0367.
45. Quasie, O., Zhang, Y.-M., Zhang, H.-J., Luo, J., and Kong, L.-Y. (2016). Four new steroid saponins with highly oxidized side chains from the leaves of *Vernonia amygdalina*. Phytochem Lett. 15, 16-20. doi: 10.1016/j.phytol.2015.11.002.
46. Ruan, J., Li, Z., Zhang, Y., Chen, Y., Liu, M., Han, L., et al. (2019). Bioactive constituents from the roots of *Eurycoma longifolia*. Molecules 24(17), 3157. doi: 10.3390/molecules24173157.
47. Saalu, L., Akunna, G., and Oyewopo, A. (2013). The Histo-morphometric Evidences of *Vernonia amygdalina* Leaf Extract-induced Testicular Toxicity. J Morphol. 31(2).
48. Saeed, R., and Shafiq, A. (2017). Effect of *Azadirachta indica* extract on phagocytic activity against the common clinical pathogen. RADS J Biol. Res Appl Sci. 8(2), 42-45.
49. Salem, M.L., and Hossain, M.S. (2000). Protective effect of black seed oil from *Nigella sativa* against murine cytomegalovirus infection. Int J Immunopharmacol. 22(9), 729-740. doi: 10.1016/S0192-0561(00)00036-9.
50. Setiawan, L.T.K., Nugraha, J., Lestari, P., Sinansari, R., Soegianto, L., Tamayanti, W.D., et al. (2019). Effect of African leaf (*Vernonia Amygdalina*) to IL-6 and IL-10 on *Staphylococcus aureus* infection. IJTID. 7(4), 6. doi: 10.20473/ijtid.v7i4.9654.
51. Shuid, A., Siang, L., Chin, T., Muhammad, N., Mohamed, N., and Soelaiman, I. (2011). Acute and subacute toxicity studies of *Eurycoma longifolia* in male rats. Int J Pharmacol. 7(5), 641-646.
52. Tambi, M.I.B.M., and Imran, M.K. (2010). *Eurycoma longifolia* Jack in managing idiopathic male infertility. Asian J Androl. 12(3), 376-380. doi: 10.1038/aja.2010.7.

53. Tran, T.T., Nguyen, N.T., Pham, N.B., Chu, H.N., Nguyen, T.D., Kishimoto, T., et al. (2018). Hairy root cultures of *Eurycoma longifolia* and production of anti-inflammatory 9-methoxycanthin-6-one. Nat Prod Commun. 13(5), 1934578X1801300507. doi: 10.1177/1934578X1801300507.
54. Tran, T.V., Malainer, C., Schwaiger, S., Atanasov, A.G., Heiss, E.H., Dirsch, V.M., et al. (2014). NF-κB inhibitors from *Eurycoma longifolia*. J Nat Prod. 77(3), 483-488. doi: 10.1021/np400701k.
55. Udeinya, I., Mbah, A., Chijioke, C., and Shu, E. (2004). An antimalarial extract from neem leaves is antiretroviral. Trans R Soc Trop Med Hyg. 98(7), 435-437.
56. Ulasli, M., Gurses, S.A., Bayraktar, R., Yumrutas, O., Oztuzcu, S., Igci, M., et al. (2014). The effects of *Nigella sativa* (Ns), *Anthemis hyalina* (Ah) and *Citrus sinensis* (Cs) extracts on the replication of coronavirus and the expression of TRP genes family. Mol Biol Rep. 41(3), 1703-1711. doi: 10.1007/s11033-014-3019-7.
57. Vahdati-Mashhadian, N., Rakhshandeh, H., and Omidi, A. (2005). An investigation on LD50 and subacute hepatic toxicity of *Nigella sativa* seed extracts in mice. Pharmazie. 60(7), 544-547.
58. Venugopalan, S.K., Viswesharan, N., Aiyalu, R., and Nair, N. (2011). Neem leaf glucosamine stimulates Interleukin-2 (IL-2) in Swiss albino mice. Nat Prec. 1-1. doi: 10.1038/npre.2011.5923.1.
59. Yee, K., Zulkawi, N., Choudhary, V.K., and Choudhary, Y.K. (2014). Evaluation of the genotoxicity of *Eurycoma longifolia* aqueous extract (Physta®) using in vitro ames test and in vivo mammalian micronucleus test. Int J Pharm Pharm. 6(4), 652-657.
60. Zakaria, Y., Azlan, N.Z., Nik, N.F., and Muhammad, H. (2016). Phytochemicals and acute oral toxicity studies of the aqueous extract of *Vernonia amygdalina* from state of Malaysia. J Med Plants Stud. 4(3), 1-5.
61. Zaoui, A., Cherrah, Y., Mahassini, N., Alaoui, K., Amarouch, H., and Hassar, M. (2002). Acute and chronic toxicity of *Nigella sativa* fixed oil. Phytomed. 9(1), 69-74. doi: 10.1078/0944-7113-00084.
62. Zihlif, M.A., Mahmoud, I.S., Ghanim, M.T., Zreikat, M.S., Alrabadi, N., Imraish, A., et al. (2013). Thymoquinone efficiently inhibits the survival of EBV-infected B cells and alters EBV gene expression. Integr Cancer Ther. 12(3), 257-263. doi: 10.1177/1534735412458827.
